# Analysing the vulnerability of mangrove forest by vegetation health assessment: a study of Indian sundarbans deltaic region

**DOI:** 10.1038/s41598-025-26905-1

**Published:** 2025-12-18

**Authors:** Amlan Ghosh, Padmaja Mondal

**Affiliations:** 1 Swami Vivekananda Institution for Teachers Training, Affiliated to Baba Saheb Ambedkar EducationUniversity, Kurmun, West Bengal 713102 India; 2 Department of Geography, Dr. Gour Mohan Roy College, Purba Bardhaman, West Bengal 713145 India

**Keywords:** Mangrove vulnerability, Vegetation indices, Vegetation health condition, Forestry, Environmental impact

## Abstract

Sundarbans region is an assemblage several delta formed in Bay of Bengal with largest concentration of mangrove which plays a crucial role in mitigating the impact of climate change with large ecosystem. The mangrove ecosystem demands further investigations to assess the vulnerability of vegetation. In context of present environmental change, the existing vegetation of Sundarbans is threatened by natural and human induced factors. This study incorporated these issues by analysing the vulnerability of mangrove forest in Indian Sundarbans deltaic region. To assess the vegetation condition, various vegetation indices are used including Normalised Difference Vegetation Index (NDVI), Transformed Normalised Difference Vegetation Index (TNDVI), Green Chlorophyll Index (GCI), Chlorophyll Vegetation Index (CVI), Soil Adjusted Vegetation Index (SAVI), and Atmospherically Resistant Vegetation Index (ARVI) etc. These indices are calculated using remote sensing satellite data of 2010 and 2020. Vulnerability has been assessed through vegetation health assessment by spatial modelling with the data from aforesaid vegetation indices. The result shows that specific regions have experienced an increase in stressed vegetation condition accompanied by the problems such as waterlogging and expanding areas under aquaculture. Furthermore the area under healthy vegetation has significantly decreased between 2010 and 2020.

## Introduction

Sundarbans deltaic region is a significant forested area with the concentration of mangrove forest that creates one of the world’s richest ecosystems^1^. Mangroves plays crucial role in protecting biodiversity by preserving the natural habitat of spices and combating the negative effects of climate change. The rich vegetation absorbs carbon di oxide, reduce coastal erosion and protect the areas along coastline from natural disaster like cyclones. Recent studies have highlighted the importance of mangrove ecosystem in disaster risk reduction. Singha et al.^2^ assessed mangrove ecosystem based disaster risk reduction in Tamil Nadu through coastal vulnerability model and found that Pichavaram and Muthupet mangrove regions were exposed to coastal hazards and loss of mangroves. Healthy mangroves along the coastline create resilience to climate change, serving as nature based adaptive measure to reduce coastal erosion and to sustain vegetation for global warming management. However, mangroves are under threat from natural and human induced factors. Sowrav et al.^3^ identified causes of mangrove decline including human intrusion, factory construction, power plant, unethical hunting, and fishing in the mangrove region. Panda et al.^4^ studied the mangrove ecosystem carbon stock in the eastern coast of India and found multiple threats like tree logging, plastic, oil and pesticide pollution, illegal prawn farming, human encroachment, land degradation from tourism development, coastal erosion, and tropical cyclone etc. Human interventions and development cause the degradation of mangrove forest emphasizing the need to study protective values of mangrove ecosystem^5^. Mangroves are managed like other forest despite their unique nature, where mangrove specific training of forest department officials can help reduce mangroves loss as studied in Bhitarkanika and Mahanadi Delta^6^. Studies have used various vegetation indices such as NDVI, to assess mangrove health. Karsch et al.^5^ found the decreased mean NDVI in the coastal part of Indian Sundarbans, while Sahana et al.^7^ explained seasonal increase in waterlogged Sundarbans due to tide and settlement area expansion. Qualitative analysis of the natural and anthropogenic drivers has been used for the vulnerability and risk assessment of the livelihood in Sundarbans^8^. The study also found that region has less economic opportunities with more dependence on ecosystem and there are continuous threats by natural hazards. The effects of climate change such as sea level change, cyclone induces storms and salinity of water and soil are visible in Sundarbans deltaic region^9,10^. Sundarbans in Bangladesh is experiencing a decline, characterised by low-lying areas, water body, and mudflat^11^. Here, Payo et al. ^12^ found that mangrove loss in Bangladesh is depended on net subsidence rates. However, some studies have challenged the notion that Sundarbans will be inundation by the end of twenty-first century, suggesting potential inundation would primarily affect barren land which lays in low elevation areas^13^. Considering the Indian and Bangladesh Sundarbans as one unit Hossain et al.^14^ identified anthropogenic factors as primary driver of decreased mangrove condition. Studies have applied remote sensing techniques to assess the mangrove health. Patra and Mishra^15^ used NDVI from Landsat 5 and Landsat 8 satellite data to assess mangrove loss and transformation in Bhitarkanika National Park along the eastern coast of India attributing human interferences. Maurya and Mahajan^16^ used hyperspectral vegetation indices like ARVI, ARI, CRI1, SIPI, PRI, NDII, PSNDb, NDNI and NDLI to estimate mangrove health in Gulf of Kutch, Gujarat. Researches on mangrove ecosystem restoration in South East Asia incorporated ecological and social attributes in around 1990s mainly in Indonesia, Thailand, Malaysia, Vietnam and Philippines^17^. In Indian Sundarbans the mangrove habitats are greatly influenced by slope and temperature^18^ and successful conservation efforts rely on local ecology and community participation^19^.

A review of existing literature reveals a gap in understanding the changing mangrove quality and connecting broader regional factors with anthropogenic drivers of mangrove loss in Indian Sundarbans. Previous studies have focussed on specific mangrove area in the deltaic part; whereas this research encompasses a larger area including Indian Sundarban forested area and surrounding C.D. Blocks as identified by Department of Sundarban Affairs. This has expanded the scope of research and allows a more comprehensive examination of anthropogenic drivers influencing vegetation condition across the region. The objectives of this study are twofold. First is to assess the vegetation condition in Indian Sundarbans and surrounded areas using different vegetation indices including NDVI, TNDVI, GCI, CVI, SAVI and ARVI. Second is to estimate vulnerability with vegetation health condition, providing a more nuanced understanding of the region’s mangrove ecosystem.

## Materials and methods

### Study area

The Sundarbans forest is enriched with richest biodiversity and United Nations Educational Scientific and Cultural Organization (UNESCO) declared the Indian portion of Sundarbans a World Heritage Site in 1987. The region is located in the southern coastal part of the state of West Bengal of India and Bangladesh where 40% of nearly 10,000 square kilometers of the Sundarban forest lies within West Bengal^1^. It is formed with several old and newly formed alluvial deposits of the marshy deltaic zone of Ganga River near Bay of Bengal. The Sundarban deltaic ecosystem serve food, river transportation, fresh water and fiber to the local and also have the externalities such as erosion management, soil retention, water and gas regulation for environmental stability by reducing natural disaster^20^. Study by Ghosh and Roy^21^ found that the younger population of this delta is coping with climate change induced loss by moving out of the region as migrant labour; this livelihood dynamics might also considered ecologically beneficial reducing anthropogenic stress on mangrove ecosystem.

**Fig. 1:**
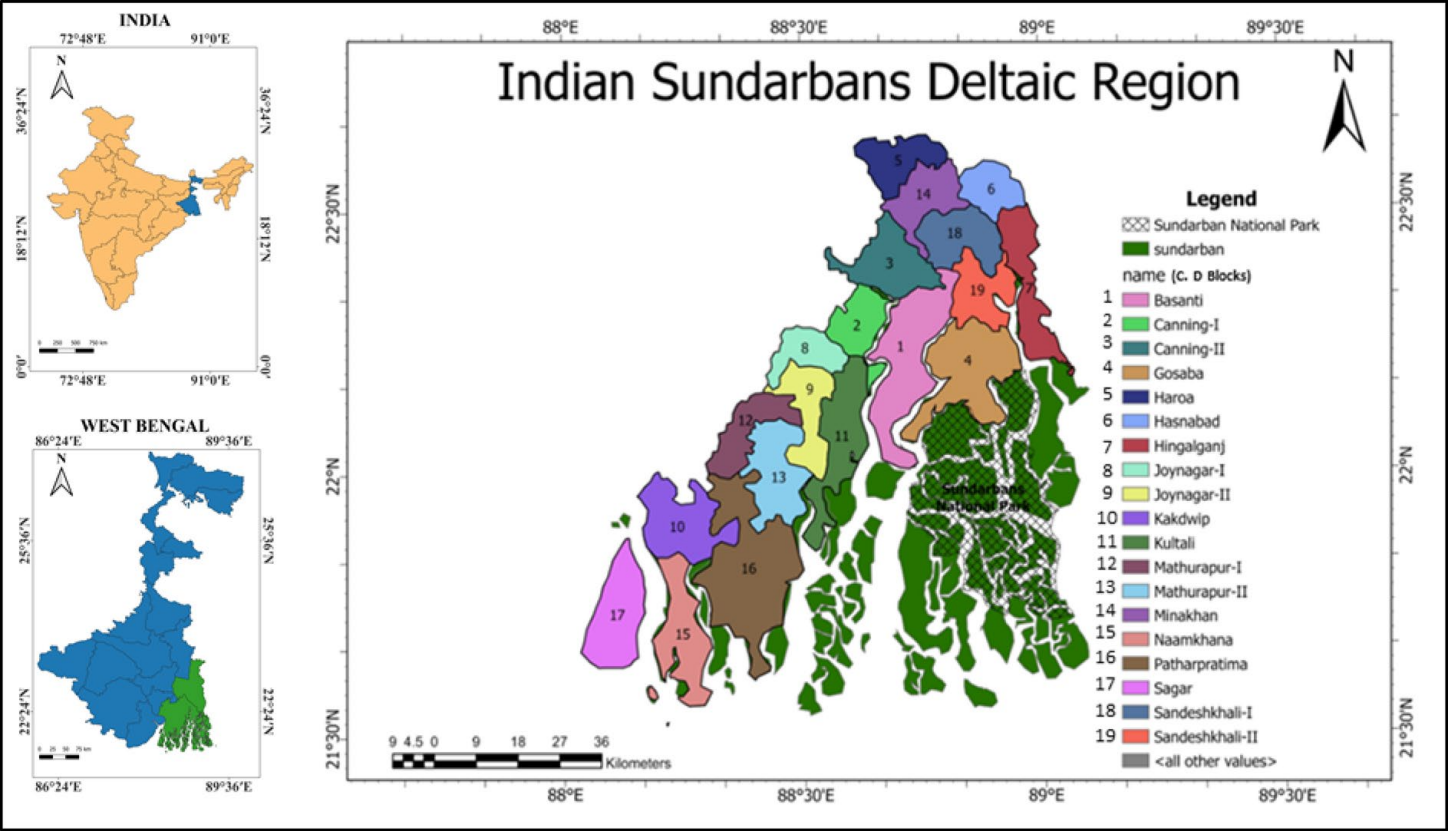
Location of the study area. Prepared from the information of Department of Sundarban Affairs, Government of West Bengal Updated On: 02 Oct 2020 (https://www.sundarbanaffairswb.in/). For administrative boundary of India and West Bengal Census of India, 2011 data have been used.

The study area of this research includes the Sundarban National Park and surrounding C.D blocks (Fig. 1). The C.D blocks have been identified from the data of Department of Sundarban Affairs, Government of West Bengal. It has been studied that Indian Sundarbans region is categorised between vulnerable to endangered with a decline in the high rates of mangrove loss; where evaluation and monitoring of the mangroves is recommended based on the hydrological modifications, sediment supply and reduction, climate change and agricultural context^22,23^. Ecosystem service valuation depicts that the quality and quantity of the mangrove ecosystem has deteriorated in last few decades with human intervention in Indian Sundarbans^24^. Sundarbans requires long term vision by integrating climate change adaptation and conservation strategies with short-term interventions like sustainable livelihood and management of human wildlife conflicts (Jana and Basu, 2022). The impact of climate change is implied over the coastal mangroves of West Bengal causing changes in mangrove quality.

### Data availability

Remote Sensing Satellite data has been used in this study for the year 2010 and 2020. The data for different bands (Blue, Green, Red and NIR) of Landsat 5 TM and Landsat 8 OLI have been collected from USGS (https://earthexplorer.usgs.gov/) for the month of December for both the years with 30 m spatial resolution (Table 1). The satellite imageries of different bands have been analyzed with QGIS Software. The data further has been modified by different vegetation indices and vegetation health assessment with spatial modeling. For the validation of the results from the analysis of satellite imageries, this study has also incorporated Google Earth historical images of specific locations along with some field visits.

**Table 1:** Data sources.

Data	Sources	Date	Path/row	Bands (wavelength in µm)	Resolution
Landsat − 5 TM	USGS	23.12.2010	138/44 and 138/45	Band 1. Blue (0.45–0.52 μm) Band 2. Green (0.52–0.60 μm) Band 3. Red (0.63–0.69 μm) Band 4. Near-Infrared (0.76–0.90 μm)	30 m
Landsat − 8 OLI	USGS	18.12.2020	138/44 and 138/45	Band 2. Blue (0.450–0.51 μm) Band 3. Green (0.53–0.59 μm) Band 4. Red (0.64–0.67 μm) Band 5 Near-Infrared (0.85–0.88 μm)	30 m

### Vegetation indices

For understanding the recent advancement in mangrove forest remote sensing data pays an important role to observe and monitor the forest^25^. Many studies used satellite data for identifying mangrove loss. Sievers et al.^23^ have addressed the stress condition of mangrove forest in Sundarbans from Sentinel-2 satellite data. The mangrove species diversity can also be delineated by Sentinel 2 A data using genus level classification with Shannon Diversity index^26^. The mangrove health status in this regard would be evaluated with the interaction of natural and anthropogenic factors considering mangrove canopy width, mangrove fragmentation, mangrove density and mangrove plant diversity as studied by Hai et al.^27^ in Mui Ca Mau of Vietnam. Chamberlain et al.^28^ studied the mangrove in Queensland, Australia where it has applied the RF classification delineating decrease in mangrove and the result has been substantiated with the mean NDVI. The coastline ecology and aftermath of cyclone can also be determined by the vegetation indices like NDVI, EVI, mVCI, DVDI studied by Mishra et al.^29,30^ in spanning of Myanmar and Bangladesh. Similar vegetation indices also have been applied in the coastal zones of Andhra and Tamil Nadu, India to assess the severe cyclonic condition along the shoreline in another study by Mishra et al.^29,30^. Vegetation indices are also useful for the determination of agricultural drought. The study by Guria et al.^31^ included thermal and optical indices like TCI, TVDI, VCI, NDDI and SMCI and microwave indices like PCI, SDCI for the Integrated Drought Severity Index (IDSI) in Odisha, India.

The following equations are used in this study for the calculations of vegetation indices from different sources (Table 2). The indices are Normalised Difference Vegetation Index (NDVI), Transformed Normalised Difference Vegetation Index (TNDVI), Green Chlorophyll Index (GCI), Chlorophyll Vegetation Index (CVI), Soil Adjusted Vegetation Index (SAVI), and Atmospherically Resistant Vegetation Index (ARVI). These indices are derived for both the year 2010 and 2020 from Landsat 5 and Landsat 8 data.

**Table 2:** Derivation of vegetation indices.

Vegetation Indices	Formula	References
1. Normalised Difference Vegetation Index (NDVI)	*NDVI = (NIR – Red)/(NIR + Red)*	Solymosi et al.^32^
2. Transformed Normalised Difference Vegetation Index (TNDVI)	*TNDVI = SQRT ((NIR– Red)/(NIR + Red) + 0.5)*	Bannari et al.^33^
3. Green Chlorophyll Index (GCI)	*GCI = (NIR)/(Green) – 1*	Nadjla et al.^3^
4. Chlorophyll Vegetation Index (CVI)	*CVI = NIR × (Red/Green* ^*2*^ *)*	Sharifi^35^)
5. Soil Adjusted Vegetation Index (SAVI)	*SAVI = ((NIR - Red)/(NIR + Red + L)) × (1 + L)*	Solymosi et al.^32^
6. Atmospherically Resistant Vegetation Index (ARVI)	*ARVI = (NIR – (2×Red) + Blue)/(NIR + (2×Red) + Blue)*	Kaufman and Tanre^36^

There are specific reasons behind selecting these six vegetation indices apart from many other indices mentioned in the literatures. NDVI has been widely used by many scholars for understanding the vegetation cover and it provide basic mangrove condition based on NIR and Red band data. TNDVI provides more accurate information with further modification in existing equation of NDVI. GCI added green band with NIR in the equation stating the chlorophyll condition in leaf which is very significant in mangrove assessment. CVI again added red band data along with NIR and green band which has significantly estimated chlorophyll content in the rich mangrove diversity. As these above applied vegetation indices do not correct the soil related noises, SAVI has been applied for getting better information about vegetation health in the areas with exposed or stress vegetation condition. Particularly in this study a larger area have been taken for better understanding of the anthropogenic factor where soil background is playing very important role for the assessment of vegetation health condition. Last ARVI has been used to minimise the atmospheric noises in the mangrove region and focussing vegetation health with forest density. The study has not included other vegetation indices like Modified Vegetation Condition Index (mVCI) and Enhanced Vegetation Index (EVI) for the analysis of mangrove health. The application mVCI is specific to the stress vegetation condition due the effect of drought, which is not significant in Sundarbans. As the atmospheric effects have been addressed by ARVI, EVI could have been given similar result. For this reason EVI has not been added for the analysis.

### Vegetation health assessment

**Fig. 2:**
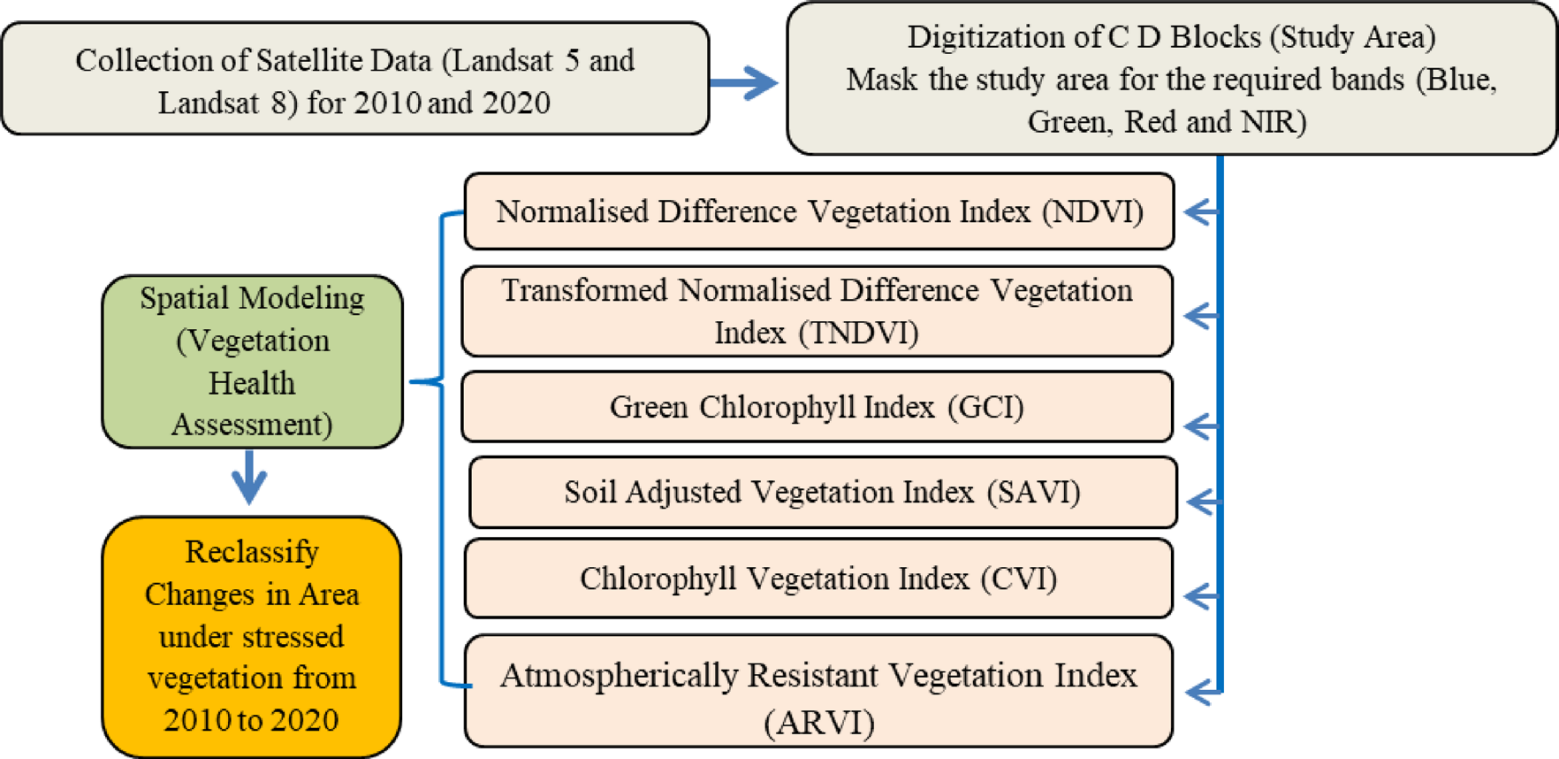
Work flow of the study.

Vulnerability of mangrove vegetation has been assessed from the satellite data by several vegetation indices and vegetation health assessment in the framework of this study (Fig. 2). Vegetation indices are used in spatial modelling for the estimation of vegetation health condition. The equation applied for that is modified from the study by Halder et al.^37^ where vegetation health condition has been calculated from NDVI, DVI, TVI, TNDVI and CVI. Halder et al.^37^ has formulated the method for addressing vegetation health condition and this study added vegetation indices such as GCI, SAVI and ARVI to the existing equation. After putting the value of indices in model maker the study has identified three categories of vegetation such as healthier vegetation, moderate vegetation and stressed vegetation.

 *Vegetation Health Condition= NDVI × TNDVI × GCI × CVI × SAVI × ARVI*.

Source: The equation is modified from Halder et al.^37^

The output from the spatial modelling is reclassified in the above categories for comparison. The areas under different vegetation conditions are also calculated to depict the changes from 2010 to 2020. The maps are prepared for all the indices for 2010 and 2020 comparing with a similar scale. The higher values represent better vegetation and the lower values depict lower concentration of vegetation. There are areas with stressed vegetation identified through spatial modelling to trace the vulnerability.

## Results

### Vegetation indices for determining health condition and vulnerability of vegetation

Indian Sundarbans forest is characterised with dense mangroves in the core area and it is getting deteriorated towards the fringe areas with the human interventions studied by different scholars. The core area is the Sundarban National Park consisting dense mangrove vegetation and the surrounding C.D blocks are characterised by less concentration of mangroves. Sahana et al.^38^ used forest canopy density by integrating the indices like average vegetation index, bare soil index, shadow index and thermal index and derived degradation in the forest canopy density in Indian Sundarbans. Pastor-Guzman et al.^39^ analysed that multispectral and hyper spectral indices best explain the variation in chlorophyll concentration in leaf and canopy. In this study it is much needed to analyse the decline in mangrove vegetation by taking different aspects like vegetation cover, chlorophyll concentration, soil and atmospheric factors etc. There are certain aspects of the mangrove vegetation that can be determined by particular indices. Pixel-based band values from Landsat 5 and Landsat 8 data are used for the calculation of several vegetation indices. The indices are focussed on the criteria of addressing particular issues on the basis of which the vegetation health is portrayed. There are small differences in the vegetation types which can be observed if all the indices are taken into consideration. The vegetation indices like NDVI only consider the NIR and Red band data which is normalized and the result is representing vegetation cover of a region. Other indices like SAVI consider the NIR and Red band data with the L factor in order to see the effect of soil colour and moisture in vegetation. The atmospherically resistance Vegetation Index (ARVI) consider the effects of the aerosol.

#### Normalized difference vegetation index (NDVI)

**Fig. 3:**
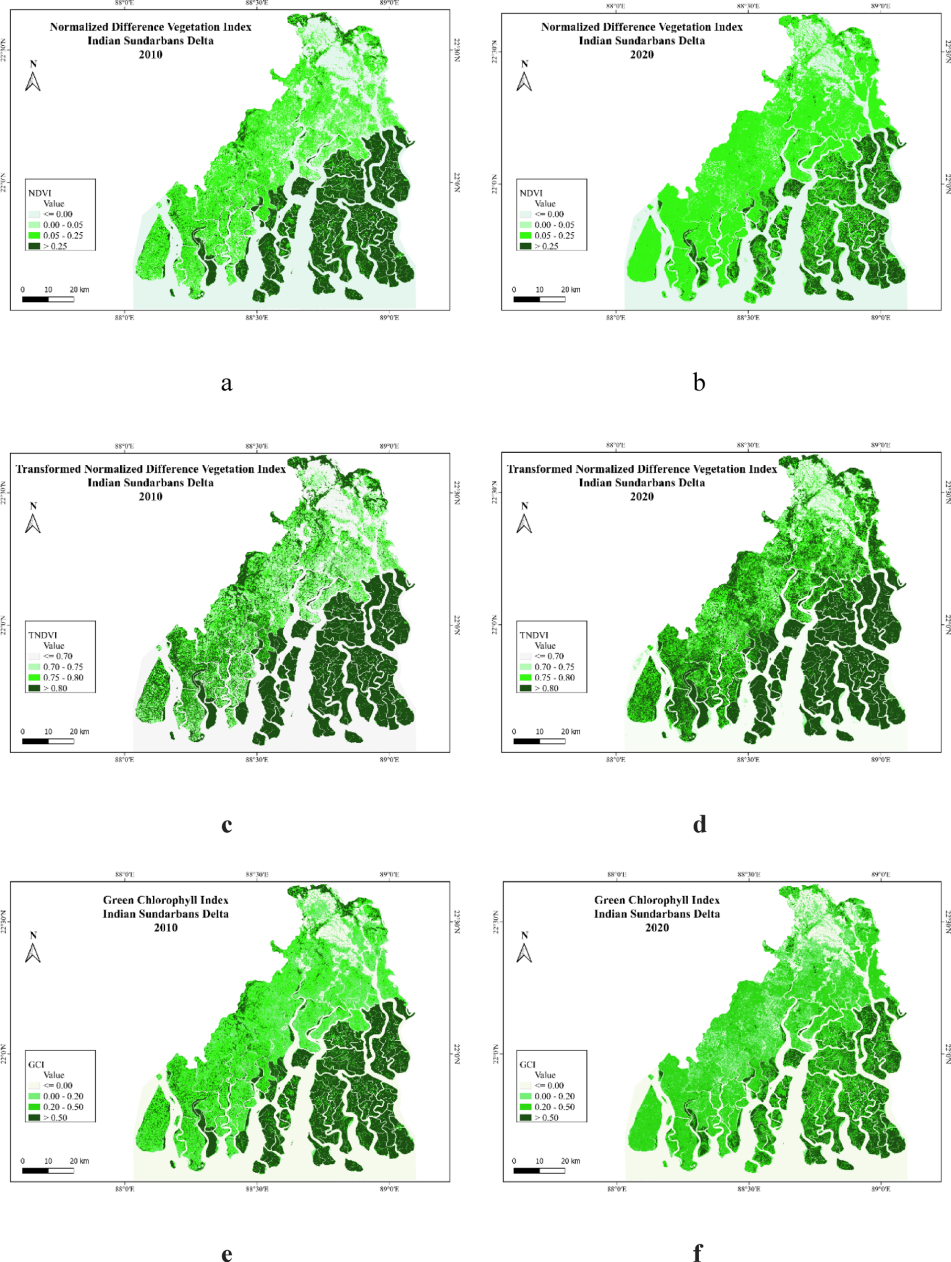

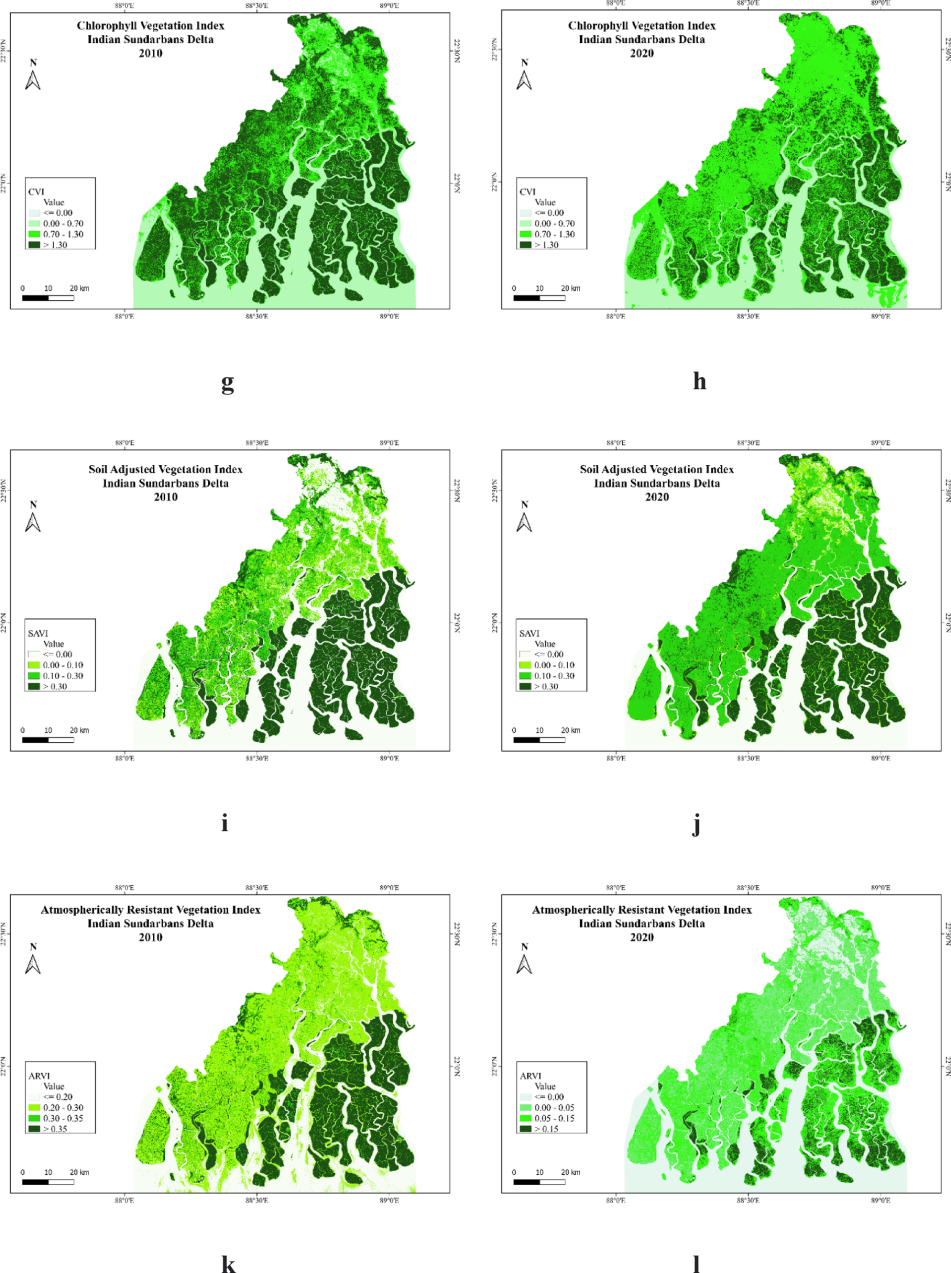
(**a**) NDVI 2010. (**b**) NDVI 2020. (**c**) TNDVI 2010. (**d**) TNDVI 2020. (**e**) GCI 2010. (**f**) GCI 2020. (**g**) CVI 2010. (**h**) CVI 2020. (**i**) SAVI 2010. (**j**) SAVI 2020. (**k**) ARVI 2010. (**l**) ARVI 2020. Prepared with QGIS version 3.18https://download.qgis.org//downloads/.

NDVI has been calculated with NIR and Red band data as these bands are exhibiting the concentration of vegetation with highest reflectance for vegetation in the spectral signature curve. The values of NDVI below zero appear as no vegetation representing the water channels of different rivers in the Indian Sundarbans deltaic region. The marshy waterlogged areas are also signifying a value below zero reflect areas with no vegetation cover. The management of mangrove using systematic approaches can be discussed further where mangrove is placed as social-ecological and integrated system with GIS based techniques and assessment of the protected areas^40^. The categorisation of vegetation has been done in this study with different range of NDVI. The values above 0.25 displays the highest concentration of mangroves in the southern and south-eastern part of the deltaic region consisting of Sundarban National Park in 2010 (Fig. 3a). There is a clear distinction between the less intensity of vegetation and deep mangrove vegetation. The changes from 2010 to 2020 articulate the decrease in the density of mangrove vegetation in the surrounding C.D. blocks and even in Sundarban National Park (Fig. 3b).

#### Transformed normalized difference vegetation index (TNDVI)

TNDVI is the modified index as derived by manipulating the NDVI with the square root and additional value of 0.5. The modified value of NDVI is thus represented by TNDVI. Highest range of the TNDVI is above 0.80 shows the deep mangroves. In the map of TNDVI for 2010 the better vegetation fragmentation is noticed even in the northern side of Sundarbans National Park like the regions of Sandeshkhali I, Sandeshkhali II, Gosaba, Hasnabad, and Haroa blocks (Fig. 3c). There are breaks in the vegetation area with marshy soil and water-logged areas in the adjacent areas of Gosaba, Matla, Bidyadhari and Ichhamati Rivers. The changes are visible from 2010 to 2020, as the vegetation concentration is increased in parts of the western side (Fig. 3d). The mangrove forest remained unchanged or slightly decreased in fragmented parts of the deltaic islands in 2020. Better concentration of vegetation is visible in TNDVI maps.

#### Green chlorophyll index (GCI)

GCI has characterised the chlorophyll content in the vegetation and it is calculated with NIR and Green band data. The indices like NDVI and TNDVI have only considered the band values of NIR and Red where the Green Chlorophyll Index (GCI) considered the value of green band which is giving a different result by showing the chlorophyll content of leafs. By comparing the GCI maps of 2010 (Fig. 3e) and 2020 (Fig. 3f), it is visible that the chlorophyll content of the vegetation decreased at a massive level. Cholophyll content is also decreased in the deep mangrove forest like Sunarban National Park in the year 2020. The rich mangrove biodiversity hotspot zones are reflecting such declines in the vegetation cholophyll content, this may indicate decreased health condition of the vegetation.

#### Chlorophyll vegetation index (CVI)

Chlorophyll Vegetation Index (CVI) is calculated with NIR, Red and Green band data. The result shows that the value of the CVI is higher than GCI in this region. The CVI map of 2010 represents the moderate to higher concentration of deep mangrove vegetation with higher chlorophyll content (Fig. 3g). The CVI concentration has been rapidly decreased in 2020 demonstrating the conversion of areas from upper to moderate class (Fig. 3h). In the previous maps of GCI, chlorophyll content changed in Sundarban National Park and surrounding islands from 2010 to 2020. But in the CVI maps, the chlorophyll content of mangroves has rapidly decreased in Sundarban National Park from 2010 to 2020 even in all the deltaic islands. The deltaic areas including Kakdwip, Sagar Island, Namkhana, and Bokkhali are also experiencing serious decline in the vegetation chlorophyll content.

#### Soil adjusted vegetation index (SAVI)

The soil adjusted vegetation index (SAVI) is a modified index where the soil colour and moisture content are considered with L factor. The moisture content and soil factor are very vital elements to analyse the health condition of the mangrove vegetation. The Sundarbans and surrounding areas are covered with marshy soil with waterlogging and deep biomass sometimes does not allow air to enter inside the pour spaces of the soil. There are breathing roots of the mangroves coming out from the soil to collect oxygen from the air. The SAVI maps are addressing the changes in vegetation where the deep mangroves are slightly modified from earlier indices (Fig. 3i). But the areas outside Sundarbans National Park changed a lot during this 10 years where the higher values are marked by better vegetation considering the soil factor (Fig. 3j). SAVI is reflecting the changes over vegetation condition in the Sandeshkhali I, Sandeshkhali II and Minakha blocks as the soil factor has controlled the vegetation with bare soil.

#### Atmospherically resistant vegetation index (ARVI)

Atmospherically resistant vegetation index has been calculated with NIR, Red and Blue band data. The result shows higher changes of mangrove vegetation in the region from 2010 to 2020. The aerosol content in the air affects the vegetation types and vegetation health condition. In the mangrove region the value of ARVI rapidly decreased from 2010 (Fig. 3k) to 2020 (Fig. 3l). The areas with less concentration of mangrove are equally affected by this process and the values are not even comparable with equal scale. The departure in the values of ARVI shows the different classes in the maps of 2010 and 2020. The causes of such changes may be impacted by the climate change and other atmospheric factors.

### Vulnerability of mangrove in Indian sundarbans

This study have analysed the vulnerability of mangrove forest with spatial modelling. The result appears that significant proportions of the northern C.D. Blocks are falling under the stressed vegetation condition. The regions like Sandeshkhali I, Sandeshkhali II, Gosaba, Basanti, Minakha and Hasnabad are serious condition in the year 2010 (Fig. 6a). The vulnerability of the vegetation in this region is also shaped by the waterlogging and soil erosion. Water channels are consisting of marshy wet lands causing erosion with depletion in mangroves and other vegetation. There are growing practices of aquaculture in the mangrove swamp and brackish water bodies. The waterlogging and storage of water during monsoon has resulted the loss of mangroves near the coast lines. The incidences of waterlogging and erosion are governed by natural disaster and it is detectable in almost all the C.D. blocks surrounding Sundarbans national park. The regions in the southern coastal part are consisting of the islands like Sagar, Namkhana, Kakdwip and Patharpratima. These are also experiencing mangrove vulnerability with increasing area under stressed vegetation.

**Fig. 4:**
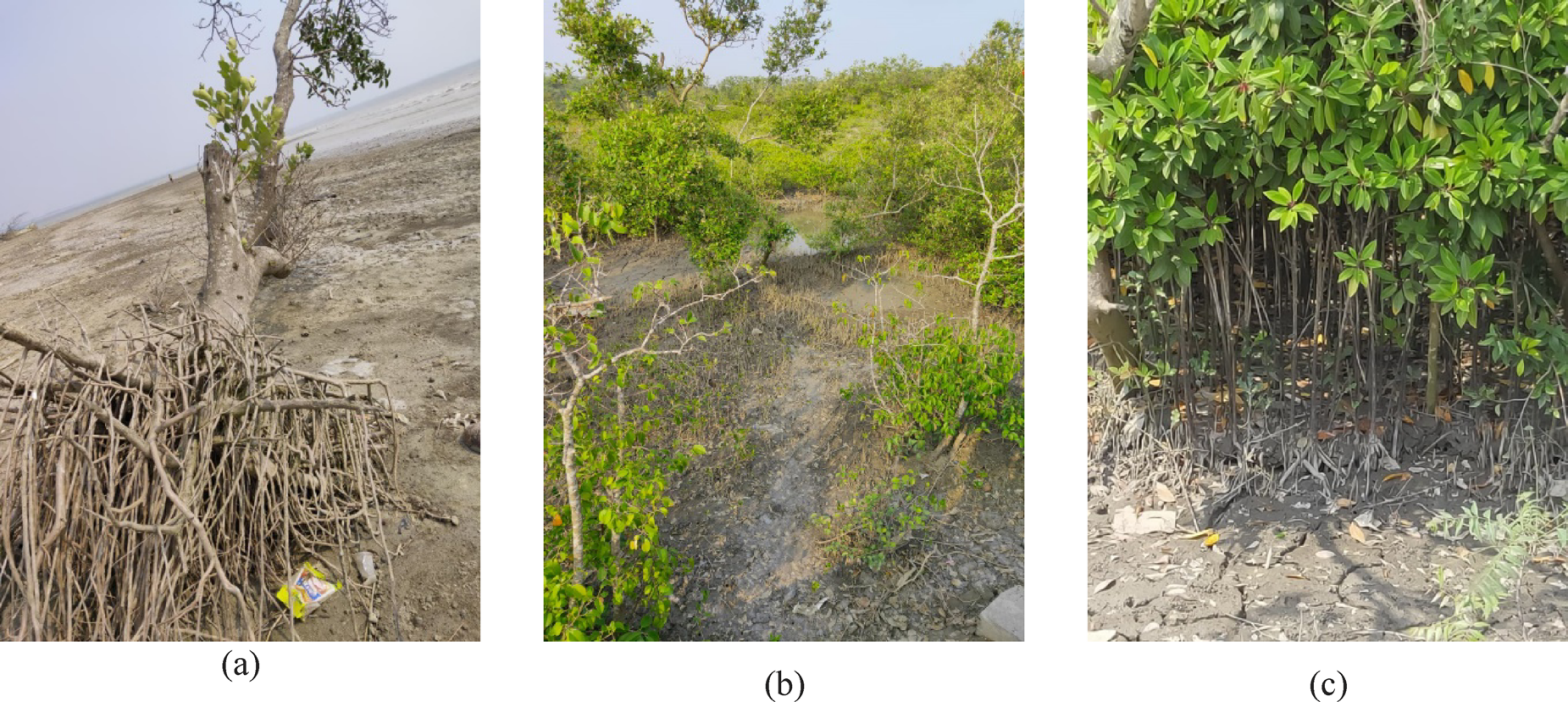
Field observations on vulnerability of mangrove vegetation in Indian Sundarbans. (**a**) Uprooting due to Stormat Mousuni Island. (**b**) Waterlogging at Henry’sIsland. (**c**) Healthy Vegetation(Sundari tree) at PatibuniaFerry Ghat.

**Fig. 5:**
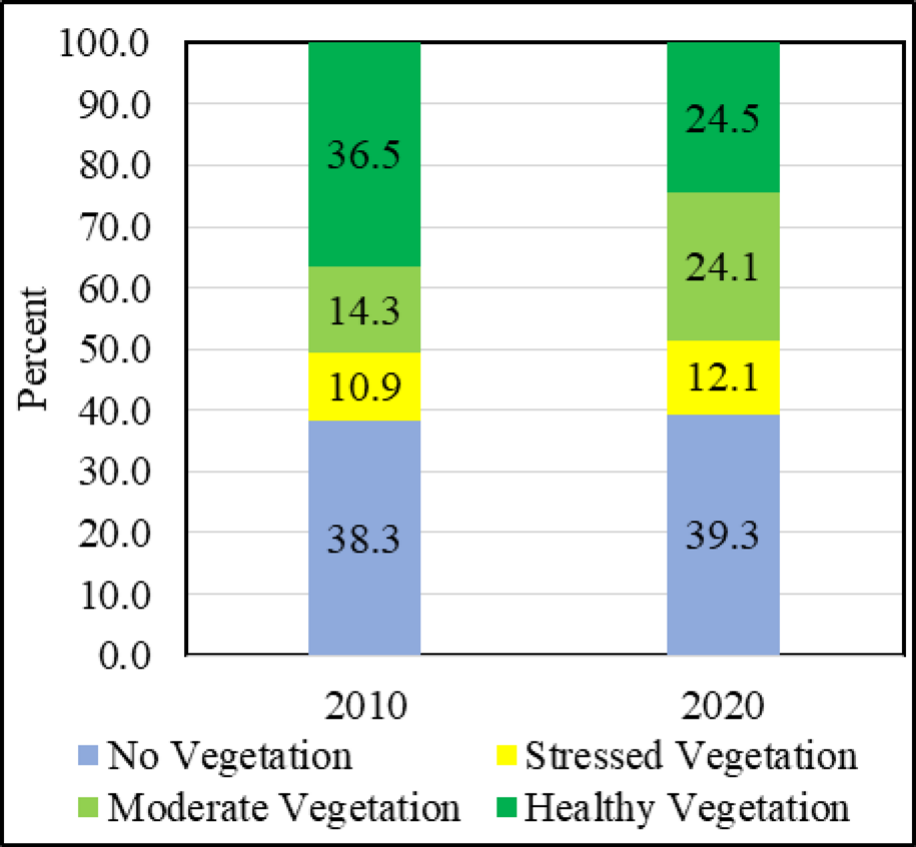
Percentage of area under different types of vegetation condition. Source: Table 3.

The vulnerability of the mangrove vegetation is increasing day by day in Indian Sundarbans as there is a significant rise in the area under stressed vegetation from 2010 to 2020. The percentage of area under stressed vegetation has increased from 10.92 to 12.13 in between 2010 and 2020 (Table 3). The frequent occurrences of natural hazards like cyclones with heavy storms and flooding resulted to the loss of mangrove and decrease in vegetation health condition (Fig. 4a). This increase in the area under stressed vegetation is also evident by the incidences of waterlogging (Fig. 4b). There is a slight increase in the area under no vegetation in the year 2020 (Fig. 5). The information has been discovered from the field that the waterlogging has given rise to the shrimp farming to a large extent in the areas like Basanti, Hingalganj, Sandeshkhali, Haroa, Minakha, Basirhat, Canning, Hasnabad and Namkhana etc. These areas are also experiencing the stressed vegetation condition. According to the local, there are practices of clearing mangroves for aquaculture in these areas which further resulted to the decrease of mangroves. From the vegetation health assessment, healthy mangroves have been identified in the Sundarbans deltaic region and some sites of surrounding areas (Fig. 4c).

**Fig. 6:**
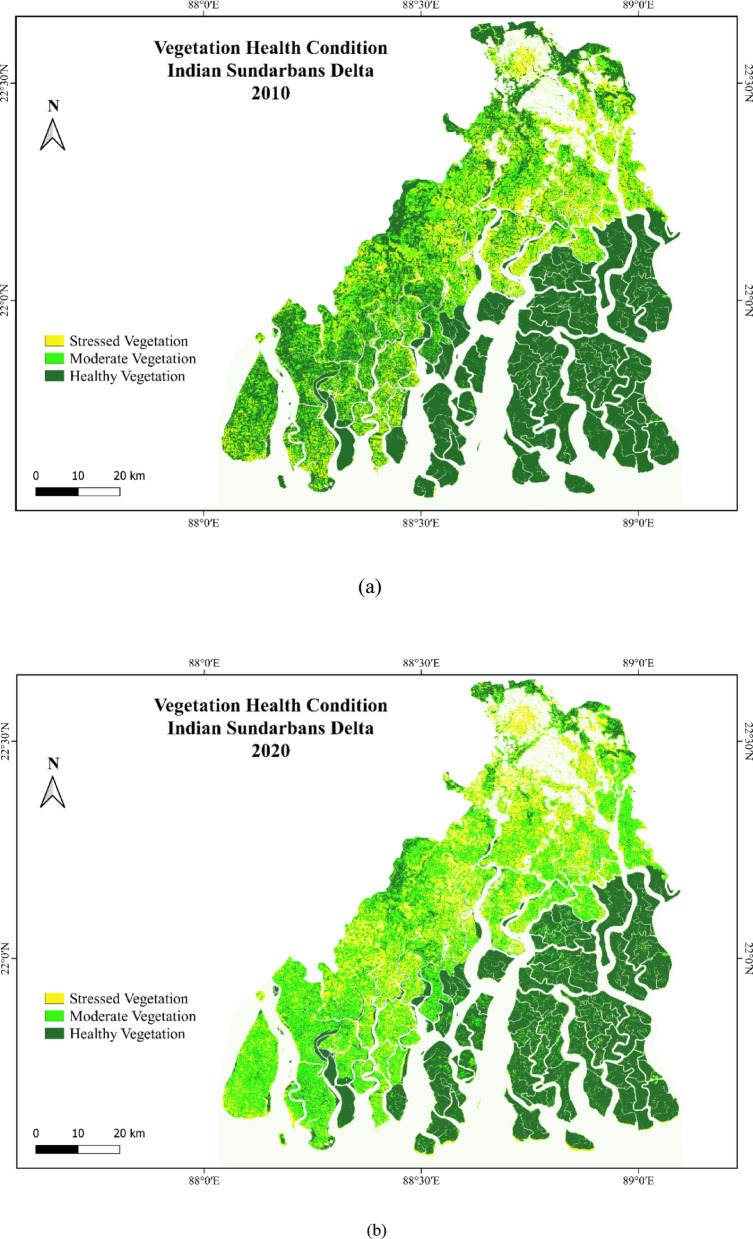

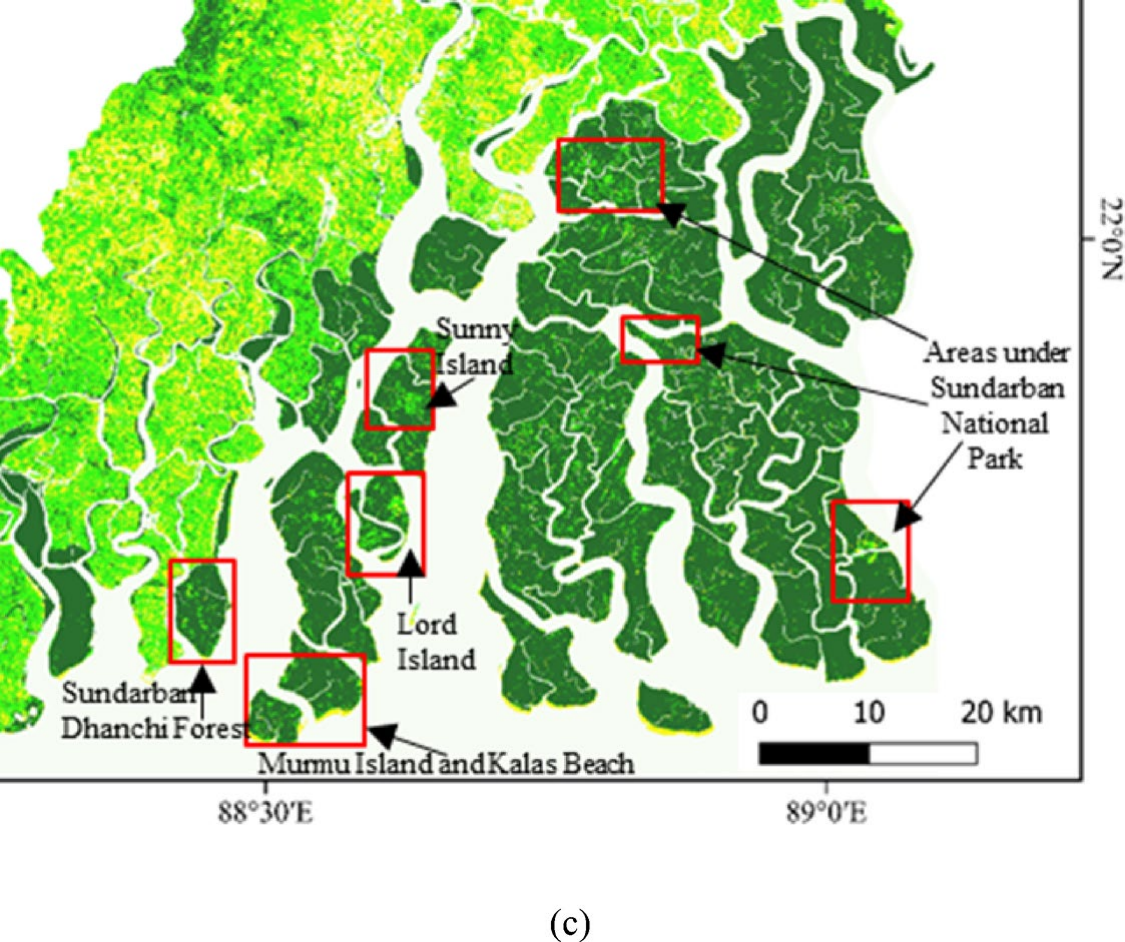
(**a**) Vulnerability of Mangrove Vegetation with assessment of vegetation health condition 2010. (**b**) Vulnerability of Mangrove Vegetation with assessment of vegetation health condition 2020. (**c**) Areas under increased stressed mangroves in the year 2020. Prepared with QGIS version 3.18 https://download.qgis.org//downloads/.

The areas with stressed vegetation have been demarcated by yellow colour which appeared in the fringe areas of Sundarbans National Park in 2010 (Fig. 6a). The extent of this area under stressed vegetation further have been increased in the map of 2020 (Fig. 6b). The fragmented mangroves with stressed vegetation are also visible in Sundarbans National Park. The national park is the reserved forest with less intimation of the human inside the forest changed a little where the fringe areas are much evident by the intrusion of the people for different purpose considering the forest wood, honey collection or tourism activity. There are certain areas which have been converted from healthy vegetation to moderate vegetation and moderate to stressed vegetation over the time. The findings of the study also directs towards the decrease of healthy vegetation from 2010 to 2020. It has been observed that percentage of areas under healthy vegetation decreased from 36.5 to 24.5 between 2010 and 2020 (Table 3). The area under healthy vegetation has been converted to moderate types of vegetation in Sundarbans. The percentage area under moderate vegetation increased from 14.1 to 24.3 between 2010 and 2020 (Fig. 5). Thus, the decrease in healthy mangrove vegetation is a serious concern for the biodiversity and environment.

**Table 3:** The area under different vegetation conditions in Indian sundarbans deltaic region.

Classes	Area in sq. km		Area in percentage	
	2010	2020	2010	2020
No vegetation	3430.44	3516.16	38.33	39.29
Stressed vegetation	977.50	1085.16	10.92	12.13
Moderate vegetation	1277.03	2153.04	14.27	24.06
Healthy vegetation	3264.69	2195.31	36.48	24.53
Total	8949.66	8949.66	100.00	100.00

Source: Derived from spatial modelling on vegetation health assessment based on the vegetation indices for 2010 and 2020.

**Fig. 7:**
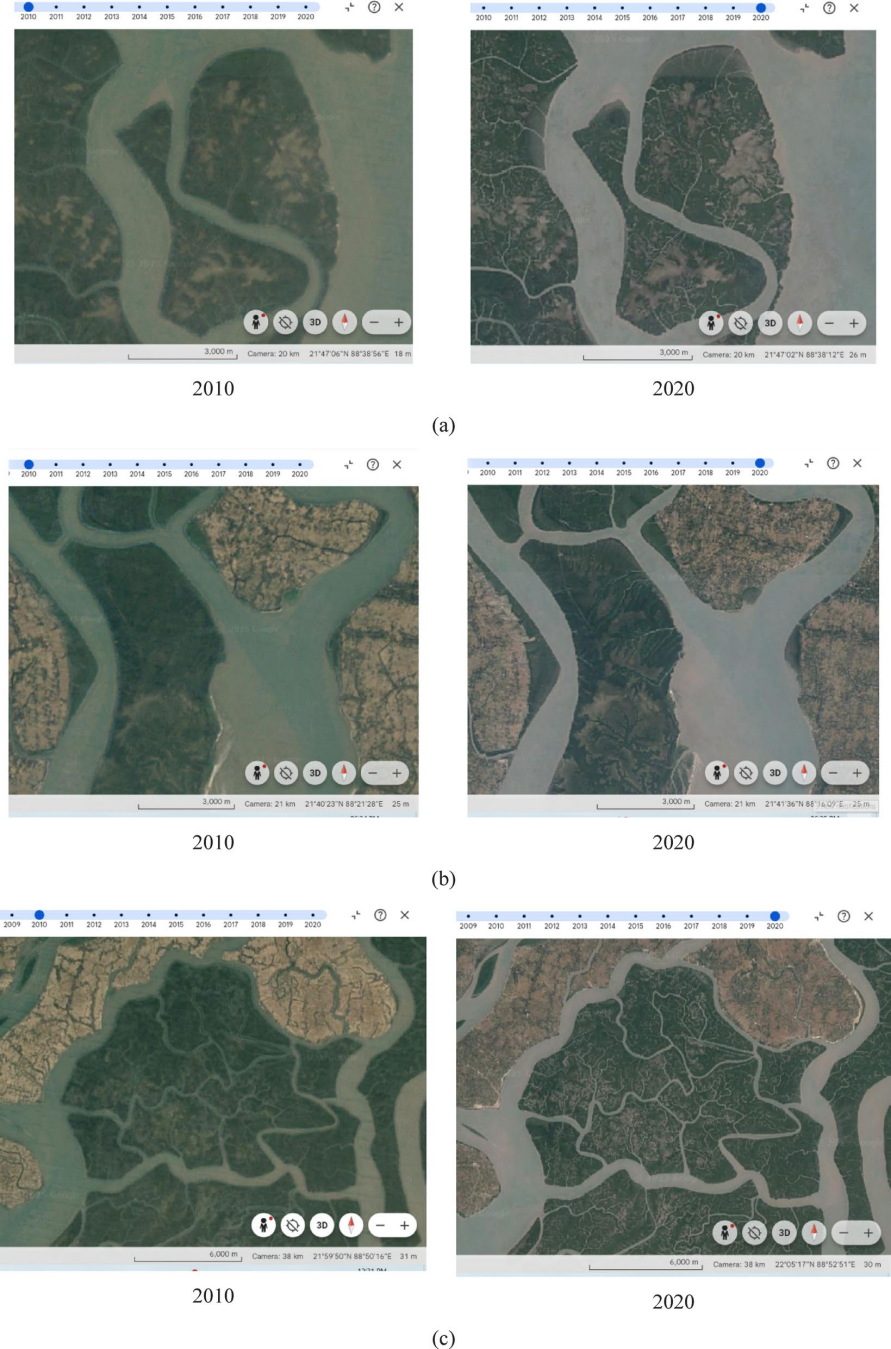
Changes in mangrove concentration from 2010 to 2020. (**a**) Changes in mangrove area due to human intrusion along Matla and Bidyadhari River (Lord Island). (**b**) Mangrove deterioration in the fringe area at Lothian Island. (**c**) River channels are causing mangrove degradation in the northern part of Sundarban National Park. Source: Google Earth historical Images (Date: 31.12.2010 and 31.12.2020).

There are pockets of mangrove deterioration in the fringe areas of Sundarban National Park and surrounding deltaic areas including Sundarban Dharchi forest, Murmu Island, Kalas Beach, Lord Island, and Sunny Island (Fig. 6c). The water channels specifically are causing the loss of mangroves due to natural processes such as tidal force, waterlogging etc. and anthropogenic drivers such as tourism and human intrusion through water channels. The decreased health condition of mangroves is supported by Google earth historical images for specific locations. The Lord Island is surrounded by Matla and Bidyadhari River channels faced decrease in mangrove health from 2010 to 2020 (Fig. 7a). This region is detached from the settlement and mostly changed in vegetation by coastal processes and soil erosion. Lothian Island is located in eastern part of Indian Sundarbans with rich mangroves, but there are pockets of decrease mangrove health condition from 2010 to 2020 (Fig. 7b). This island is very near to the popular tourist places like Namkhana and Bokkhali and used as a tourist destination at Lothian Wildlife Sanctuary. The decrease health condition of mangroves is caused by natural as well as anthropogenic factors. The northern part of Sundrban National Park is surrounded by the water channels of Bidyadhari River. There are tourism activities growing along the boundary of Sundarban National Park along sides of Bidyadhari River comprising different eco-resorts. Mangrove health condition also has been decreased from 2010 to 2020 in this region (Fig. 7c).

## Discussion

The study analyses vulnerability of mangroves vegetation in Sundarban National Park and surrounding C.D Blocks with its ecological significance and rich biodiversity. The deltaic region is showing greater concentration of mangroves with the formation of estuaries and multiple water channels which are flooded by tidal water. Mangroves and other types of vegetation are largely influenced by these estuaries in Sundarbans and surrounding areas. The natural and anthropogenic factors are affecting the vegetation condition in Sunadrbans^5,18^. The natural factors are soil erosion, tidal energy, sea level rise and climate change and the anthropogenic factors are human intervention by fishing, factories, unethical hunting^3^. This study has discussed the vegetation health condition in the region by different vegetation indices and spatial modelling. The findings have pointed out a decrease in quality of vegetation in this region where NDVI and other indices show a decline from 2010 to 2020. This is quite similar from the findings of earlier studies as there are concerns about climate change and other anthropogenic factors influencing mangrove ecosystem. Comparing different vegetation indices, it has been found that TNDVI has given better differentiation than NDVI to discriminate the mangroves types. GCI and CVI both the indices have clarified the chlorophyll content in the deltaic vegetation. GCI has well-articulated the decrease in chlorophyll concentration specific to the deltaic mangrove region and CVI on the other hand has shown serious decrease in chlorophyll concentration of the vegetation in whole region. CVI in this case has given better differentiation in chlorophyll content in the whole region including mangroves and non-mangroves vegetation. CVI has added red band data in addition to the NIR and green band which has resulted to specific index value. Rich biodiversity in the region has produced higher quantity of hummus in the soil from the decayed bodies of vegetation and animals. There is dark soil in this area which is characterised with low pour spaces as there are presence of mud and hummus in the soil of deltaic types. The indices like NDVI, TNDVI, GCI and CVI have not included soil related issues. So, SAVI has been applied to identify the effect of soil factor in the vegetation. The result shows that the vegetation of surrounding C.D. blocks around Sundarbans has been largely affected with the waterlogging and soil erosion as visible in Sandeshkhali I, Sandeshkhali II and Minakha. To address the atmospherically issues such as aerosol content, ARVI has been applied incorporating blue band data in addition to NIR and Red. Comparing the result with other vegetation indices, it has been noticed that mangrove vegetation condition even declined in Sundarban National Park.

Considering the ecological factors like vegetation extent, chlorophyll content, soil and aerosol the study have determined a serious concern over decline in the vegetation condition of Indian Sundarbans. The health assessment of the vegetation shows the stressed vegetation in Sundarban forest and surrounding areas are caused by both natural and anthropogenic reasons. There is an increasing pattern in human interventions such as fisheries have largely affected the surrounding areas. As specified by Banerjee et al.^8^ and Jamal et al.^41^ the anthropogenic drivers are always causing serious threat to the natural vegetation, this study has witnessed the similar pattern. There are human intrusions through multiple water-channels due to tourism activities and in the specifics Islands with permanent arrangements like camps causing decrease in mangrove condition in the deltaic deep vegetation. On the other side, the increase areas under shrimp cultivation has removed the vegetation in the areas like Basanti, Hingalganj, Sandeshkhali, Haroa, Minakha, Basirhat, Canning, Hasnabad and Namkhana etc. The natural waterlogging and soil erosion by tidal water caused a serious damage over the vegetation in this region and human activities in addition to this reduced the intensity and quality of vegetation.

## Policy implications

The region may adopt long term measures focusing on community based participation for mangrove restoration and prevention of climate change impacts. The high risk of decreasing mangrove health can also be addressed by building resilience and strategic ecological management. There fringe areas of Sundarban forest should be protected by limiting the tourism related activities such as building hotels, camps and boating in water channels. Government regulation in tourism related activities should be effectively applied to sustain the natural environment and training of official should be done specific to mangrove forest. Participation of the local communities based on traditional knowledge system can be activated for the restoration of stressed vegetation. The population of surrounding regions are dependent on Sundarbans by many ways for their livelihood. The management framework should also secure the livelihood of local population and natural environment. Many NGOs are working in Sundarbans by planting the mangroves in the deforested tracks involving the local people. The arena of such initiatives should be broadened by government support and policy formulation.

## Conclusion

In context of global concerns over mangrove loss in different parts of the world, this study of Indian Sundarbans reflects a specific pattern where human–nature interactions are causing threat to the natural ecosystem. The vegetation indices like NDVI, TNDVI, GCI, CVI, SAVI and ARVI have clarified different aspects of mangroves and other vegetation in the deltaic estuaries like extent of vegetation, chlorophyll content, soil factor and atmospheric effect etc. The changes from 2010 to 2020 show a sharp decrease in chlorophyll content of mangroves as well as soil factors and atmospheric effects on vegetation. This study only has incorporated the vegetation indices in the spatial modelling for the assessment of vegetation health condition. The results showed that area under healthy vegetation decreased by 11.95% from 2010 to 2020 with an increase in area under stressed vegetation by 1.21% in the study region. The surrounding C.D. blocks of Sundarban forest have less concentration of mangroves and this is even declined from 2010 to 2020. The northern C.D. Blocks like Sandeshkhali I, Sandeshkhali II, Gosaba, Basanti, Minakha, Hasnabad and several others regions are facing the problem of water logging due to lower elevation and coastal vulnerability that resulted to the depletion of mangrove. Sundarban National Park consisting of several deltaic islands and surrounding islands is showing higher concentration of mangroves but it is also declined over time as visible in decreased chlorophyll content. Human activities specific to aquaculture in shrimp cultivation are rapidly growing by removing mangroves in the areas like Basirhat, Canning, Hasnabad and Namkhana etc. The study should be further extended by including the factors like extent of waterlogging, sea level changes, land surface temperature, carbon stock, livelihood mechanism etc. These would lead towards the assessment of species specific loss of mangroves and points of human interaction for sustainable management.

## Limitations

The study has determined mangrove vulnerability on the basis of mangrove health condition using spatial modelling. There are other methods of analysing the vulnerability of mangrove by incorporating different natural and human induced factors affecting extent and quality of mangroves. Although the study has found such factors in explaining the reasons behind decrease in mangrove health condition in Indian Sundarbans. There are some instances of field verification in areas under stressed and healthy vegetation but the data is only used for finding the reasons behind such decline in the mangrove health and not used statistically for quantitative accuracy assessment.

## Data Availability

Remote Sensing Satellite data has been used in this study for the year 2010 and 2020. The data for different bands (Blue, Green, Red and NIR) of Landsat 5 TM and Landsat 8 OLI have been collected from USGS (https://earthexplorer.usgs.gov/) for the month of December for both the years with 30 m spatial resolution (Table 1). The satellite imageries of different bands have been analyzed with QGIS Software. The data further has been modified by different vegetation indices and vegetation health assessment with spatial modeling. For the validation of the results from the analysis of satellite imageries, this study has also incorporated Google Earth historical images of specific locations along with some field visits.
